# Lymphocytes Contribute to the Pathophysiology of Neonatal Brain Injury

**DOI:** 10.3389/fneur.2018.00159

**Published:** 2018-03-19

**Authors:** Arshed Nazmi, Anna-Maj Albertsson, Eridan Rocha-Ferreira, Xiaoli Zhang, Regina Vontell, Aura Zelco, Mary Rutherford, Changlian Zhu, Gisela Nilsson, Carina Mallard, Henrik Hagberg, Jacqueline C. Y. Lai, Jianmei W. Leavenworth, Xiaoyang Wang

**Affiliations:** ^1^Department of Neuroscience and Physiology, Sahlgrenska Academy, University of Gothenburg, Gothenburg, Sweden; ^2^Department of Clinical Sciences, Sahlgrenska University Hospital, Gothenburg, Sweden; ^3^Department of Pediatrics, The Third Affiliated Hospital of Zhengzhou University, Zhengzhou, China; ^4^Henan Key Laboratory of Child Brain Injury, The Third Affiliated Hospital of Zhengzhou University, Zhengzhou, China; ^5^Department of Perinatal Imaging and Health, Centre for the Developing Brain, King’s College London, St. Thomas’ Hospital, London, United Kingdom; ^6^Department of Neuroscience and Physiology, Center for Brain Repair and Rehabilitation, Sahlgrenska Academy, University of Gothenburg, Gothenburg, Sweden; ^7^Department of Neurosurgery, University of Alabama at Birmingham, Birmingham, AL, United States; ^8^Department of Microbiology, University of Alabama at Birmingham, Birmingham, AL, United States

**Keywords:** lymphocytes, preterm, brain damage, mouse models, hypoxia–ischemia, brain

## Abstract

**Background:**

Periventricular leukomalacia (PVL) is the most common form of preterm brain injury affecting the cerebral white matter. This type of injury involves a multiphase process and is induced by many factors, including hypoxia–ischemia (HI) and infection. Previous studies have suggested that lymphocytes play a significant role in the pathogenesis of brain injury, and the aim of this study was to determine the contribution of lymphocyte subsets to preterm brain injury.

**Methods:**

Immunohistochemistry on brain sections from neonatal mice was performed to evaluate the extent of brain injury in wild-type and T cell and B cell-deficient neonatal mice (*Rag1*^−/−^ mice) using a mouse model of HI-induced preterm brain injury. Flow cytometry was performed to determine the presence of different types of immune cells in mouse brains following HI. In addition, immunostaining for CD3 T cells and CD20 B cells was performed on postmortem preterm human infant brains with PVL.

**Results:**

Mature lymphocyte-deficient *Rag1*^−^*^/^*^−^ mice showed protection from white matter loss compared to wild type mice as indicated by myelin basic protein immunostaining of mouse brains. CD3^+^ T cells and CD20^+^ B cells were observed in the postmortem preterm infant brains with PVL. Flow cytometry analysis of mouse brains after HI-induced injury showed increased frequency of CD3^+^ T, αβT and B cells at 7 days after HI in the ipsilateral (injured) hemisphere compared to the contralateral (control, uninjured) hemisphere.

**Conclusion:**

Lymphocytes were found in the injured brain after injury in both mice and humans, and lack of mature lymphocytes protected neonatal mice from HI-induced brain white matter injury. This finding provides insight into the pathology of perinatal brain injury and suggests new avenues for the development of therapeutic strategies.

## Introduction

Brain injury in premature infants born at less than 30 weeks gestational age is a significant clinical problem (1–3). Many important maturation processes occur during the last half of gestation, including the development of premyelinating oligodendrocytes (pre-OLs), axons, and neurons (4). These events are complex and rapid, and they are, therefore, vulnerable to endogenous and exogenous insults such as inflammation, decreased blood flow (ischemia), decreased oxygen flow (hypoxia), and free radical activity (1).

Periventricular leukomalacia (PVL) is generally thought to be the most common form of injury to the preterm brain. PVL is a distinct form of cerebral white matter injury that is characterized by the loss of pre-OLs and is associated with a high risk of neurodevelopmental impairment (1, 2, 5). Cerebral ischemia, maternal infections, and fetal systemic inflammation are the primary factors that initiate PVL through excitotoxicity and the production of free radicals (6). Preterm hypoxia–ischemia (HI) is thought to be one of the leading causes of brain injury secondary to maternal infection (7), and infection of the chorioamniotic membrane with pathogenic bacteria (chorioamnionitis) is considered to be a life-threatening risk factor for preterm infants because it can directly cause brain injury and can make the fetal brain more vulnerable to insults such as hypoxia (8, 9).

The exact role of inflammation in neonatal brain injury is still not fully understood, although it is known that sterile inflammation is associated with the recruitment of peripheral immune cells to the brain. Early infiltrating cells after insult to the brain include polymorphonuclear leukocytes and monocytes, and lymphocytes can also enter in both the neonatal (10–14) and adult brain following ischemia. In the damaged brain, these cells release proinflammatory molecules such as those involved in type 1/type 17 immune responses (15) and anti-inflammatory cytokines that can either aggravate or repair injury (16, 17).

Understanding the dynamics of postischemic inflammation is a prerequisite for therapeutic intervention in this fragile system in order to prevent harmful side effects of such interventions in sick neonates. Here, we hypothesize that lymphocytes can gain access into the brain after HI-induced brain injury and contribute to the development of neonatal brain injury. The purpose of this study was (1) to determine the presence of lymphocytes in the neonatal mouse brain in our established mouse model of HI-induced preterm brain injury using postnatal day (PND) 5 neonatal mice (15) and (2) to evaluate the contribution of mature T and B cells to neonatal brain injury.

## Materials and Methods

### Animals

C57Bl/6J wild-type (WT) and recombination-activating gene 1 (*Rag1*) mutant mice (*Rag1*^−^*^/^*^−^; B6.129S7-Rag1^tm1Mom^/J) were purchased from Jackson Laboratories (Bar Harbor, ME, USA) and were bred in the animal facility at the University of Gothenburg (Experimental Biomedicine, University of Gothenburg). Mice were housed with a 12-h light/dark cycle and had free access to a standard laboratory chow diet (B&K, Solna, Sweden) and drinking water. All animal experiments were carried out in accordance with the recommendations of the Animal Ethical Committee of the University of Gothenburg, which approved the protocol (ethical number 5/2013 and 58-2016).

### HI Procedure

For flow cytometry and immunohistochemical staining, WT and *Rag 1*^−/−^ mice of both sexes were exposed to HI at PND5, a developmental stage where the brain development in the animals corresponds to preterm infants. The period PND2–5 is when rodents have the highest percentage of pre-OLs and thus mimic the vulnerable developmental stages of preterm human infants (18). Briefly, mice were anesthetized with isoflurane (5.0% for induction and 1.5–3.0% for maintenance). The left common carotid artery was ligated, and the mice were returned to their dam and allowed to recover for 1 h. The mice were then placed in an incubator perfused with a humidified gas mixture (10% oxygen) at 36°C for 70 min. After HI, the pups were returned to their dam until they were sacrificed. For flow cytometry, WT mouse pups were sacrificed at 6 h, 3 days, or 7 days after HI, and for immunohistochemical staining, both WT and *Rag 1*^−/−^ mice were sacrificed 7 days after HI.

### Assessment of Brain Damage

Deeply anesthetized mouse pups were subjected to transcranial perfusion with saline and 5–10% buffered formaldehyde (Histofix; Histolab Products AB, Gothenburg, Sweden). Dissected mouse brains were embedded in paraffin and cut into 10-µm coronal sections. The extent of white matter and gray matter injury at the hippocampal level was analyzed after immunohistochemical staining for myelin basic protein (MBP) and microtubule-associated protein 2 (MAP-2), respectively. The quantitative measurements of the brain injury were done by manually outlining the MBP- and MAP-2-positive areas usingMicro Image version 4.0 (Micro-macro AB, Gothenburg, Sweden) as previously described (9) by an investigator blinded to the treatment groups.

We measured the following three parameters for each brain: (1) the total MAP-2-positive area in both brain hemispheres, (2) the total MAP-2-positive area in the hippocampus area of both hemispheres, and (3) the MBP-positive area in the subcortical white matter in both hemispheres. The total tissue loss was calculated as: (Contralateral hemisphere − Ipsilateral hemisphere)/(Contralateral hemisphere) × 100%. One brain section/mouse at the hippocampus level was evaluated. Our previous study using simple linear regression analysis comparing the total brain tissue loss (volume) with the tissue loss in one representative brain section (area) from the hippocampus level showed a significant positive linear correlation between the two methods (15), suggesting that a single representative brain section/mouse can be used to estimate tissue loss.

### Human Postmortem Brains

Informed parental consent was acquired from all individual participants included in the study according to the World Medical Association’s Declaration of Helsinki and the guidelines of the National Health Service UK. Ethics permission for the study was obtained from the National Research Ethics Service, Hammersmith and Queen Charlotte’s and Chelsea Research Ethics Services, London, UK (ethics number 07/H0707/139). Three extremely preterm postmortem brains (<28 weeks gestational age) of vaginally delivered neonates with PVL, and three without PVL (serving as controls), were obtained from the Department of Perinatal Pathology, Imperial Health Care Trust, London, UK. Paraffin-embedded tissue sections from the frontal and parietal lobes (at the level of Ammon’s horn) of the postmortem brains were used for immunohistochemistry staining. The primary cause of death of each case was assessed by a pathologist. The details of each case are summarized in Table 1.

**Table 1:** Summary of the case data for human postmortem brain immunohistochemistry analysis.

Case	PVL/control	GA at birth (weeks)	Postnatal age	Case details	IHC staining: periventricular white matter	IHC staining: meninges
1	Control	23 + 6/7	9 h 51 min	No brain pathology observed. Male, 545 g Sepsis, cefotaxime treatment	CD3: – CD20: –	CD3: – CD20: –
2	Control	27 + 5/7	<1 day	No brain pathology observed. Female, 886.4 g Atrioventricular septal defect	CD3: ± CD20: –	CD3: ± CD20: –
3	Control	28 + 1/7	<1 day	No brain pathology observed. Male, 1308.4 g No infection, no antibiotics	CD3: ± CD20: ±	CD3: ± CD20: ±
4	PVL	26 + 5/7	1 day 7 h	PVWMI. Male. 641.4 g Oligohydramnios, maternal hypertension	CD3: + CD20: +	CD3: ++ CD20: +
5	PVL	26 + 6/7	2 days	PVWMI. Male, 807.6 g Patent ductus arteriosus	CD3: + CD20: +	CD3: ++ CD20: +
6	PVL	24 + 6/7	16 days 19 h	PVWMI. Male, 715.4 g Rupture of membranes; oligohydramnios, AFI	CD3: + CD20: +	CD3: + CD20: +

*−: no positive cells; ±: positive cells seen inside the blood vessels; +: positive cells seen; + +: positive cells easily seen*.

*GA, gestational age; PVL, periventricular leukomalacia; PVWMI, periventricular white-matter injury; AFI, amniotic fluid infection*.

### Immunohistochemistry Staining

Immunohistochemistry procedures were performed as previously described (10). Mouse brain sections were immersed in xylene twice and then in graded ethanol (100, 95, and 70%) to remove the paraffin and rehydrate the brain sections. Antigen retrieval was then performed by boiling the sections in sodium citrate buffer (pH 6.0) for 10 min followed by 3% H_2_O_2_ for 10 min to block endogenous peroxidase activity and background staining. After antigen recovery and blocking, the sections were incubated overnight at 4°C with the mouse anti-MAP-2 (clone HM-2, Sigma-Aldrich, Stockholm, Sweden) or mouse anti-MBP (SMI94, Covance, NJ, USA) primary antibodies followed by incubation with the appropriate biotinylated secondary antibody (Vector Laboratories, Burlingame, CA, USA) for 1 h at room temperature. Visualization was performed using Vectastain ABC Elite with 3,3′-diaminobenzidine (DAB) enhanced with ammonium nickel sulfate, beta-d glucose, ammonium chloride, and beta-glucose oxidase (all from Sigma-Aldrich, Stockholm, Sweden).

The processing of the human postmortem brains and the immunohistochemical staining were performed as described previously (19, 20). Human brain sections were blocked in 5% goat serum (Vector Laboratories) for 20 min before being incubated overnight at 4°C in a solution of anti-human CD3 antibody (cat. Code A0452, clone F7.2.38, Agilent, Carpento, CA, USA) or anti-CD20 antibody (cat. MA5-13141, clone L26, Agilent). The next day, the sections were incubated with biotinylated goat anti-rabbit IgG secondary antibody (15 µg/ml; Vector Laboratories) in PBS for 1 h followed by avidin–biotin complex for 1 h (1:200 dilution; Vector Laboratories). The reactions were visualized with DAB (Sigma-Aldrich, Gillingham Dorset, UK) for 10 min. Finally, the sections were dehydrated, cleared in xylene, and coverslipped. As negative controls, we performed staining in the absence of the primary antibodies and instead used the isotype controls of the respective primary antibodies. The sections were examined under bright-field microscopy using a light microscope (DM6000 B; Leica Microsystems Ltd., Bucks, UK).

### Mouse Brain Mononuclear Cell (MNC) Isolation and Flow Cytometry Analysis

Mice were sacrificed at 6 h, 3 days, or 7 days after HI for flow cytometric analysis. After a brief transcranial perfusion with saline, the brains were dissected out and divided into ipsilateral and contralateral hemispheres. To prepare single-cell suspensions, the dissected brains were cut into small pieces using a sharp razor blade and incubated with an enzyme mixture containing 0.01% papain, 0.01% DNase I (Worthington, NJ, USA), 0.1% Dispase II (Roche, Sweden), and 12.4 mM MgSO_4_ in Ca/Mg-free HBSS (Thermo Fisher, Sweden) for 20 min at 37°C with gentle triturations before incubation and after 10 min of incubation. The single-cell suspensions were centrifuged at 300 *g* for 5 min at 4°C, and the pellets were resuspended in 5 ml of 30% isotonic Percoll (Amersham Biosciences) that was overlaid onto 70% isotonic Percoll. The Percoll gradient was centrifuged at 1,000 *g* for 30 min at room temperature. MNCs were collected from the 70/30% Percoll interface and washed with ice-cold PBS containing 1% bovine serum albumin.

In order to identify adaptive immune cells in brain samples, isolated MNCs (1 × 10^6^ cells) from each sample were first incubated with anti-mouse CD16/32 antibody in 100 µl FACS buffer (PBS and 1% BSA) for 15 min at 4°C to block the Fc receptor and then stained with anti-CD3 (FITC, 145-2C11, BD Biosciences), anti-TCRαβ (APC Cy™7, clone H57-597, BD Biosciences), anti-TCR γδ (PE-Cy™7, clone GL3, eBioscience), anti-CD19 (PE, clone 1D3, BD Biosciences), anti-CD45 (V500, clone 30-F11, BD Biosciences), and anti-CD11b (APC, clone M1/70, eBioscience) antibodies for 30 min at 4°C. After staining, the samples were washed with 500 µl FACS buffer, and the pellets were resuspended in 350 µl of FACS buffer. Dead cells were labeled by adding 7-AAD to the final 10 min of antibody staining, and these cells were excluded from the analysis. All samples were immediately acquired on a BD FACSCantoII™ flow cytometer. A total of 5 × 10^5^ events was acquired, and the data were analyzed with FlowJo software (Tree Star, Ashland, OR, USA).

Cells were first gated based on size and granularity, then doublets and dead cells were excluded. At the 6 h time point where CD45 and CD11b were included in the staining cocktail, CD3^+^, CD19^+^, CD3^+^, and αβTCR^+^ lymphocyte population back gating on the CD45 and CD11b plot was performed to ensure that they were CD45 single-stained cells and that the identified cell populations were not the result of nonspecific binding of antibody to cells of myeloid origin with incomplete Fc blockage (Figures S1 and S2 in Supplementary Material).

### Statistics

Data were analyzed by Student’s paired *t*-test (for flow cytometry data) and Student’s unpaired *t*-test (for the tissue loss evaluation data), and the results are presented as means ± SEM with significance set at *p* < 0.05. All statistical analyses were carried out using GraphPad Prism software (version 6.02).

## Results

### CD3^+^ T and CD20^+^ B Cells Were Found in the Postmortem Preterm Infant Brains With PVL

**Figure 1:**
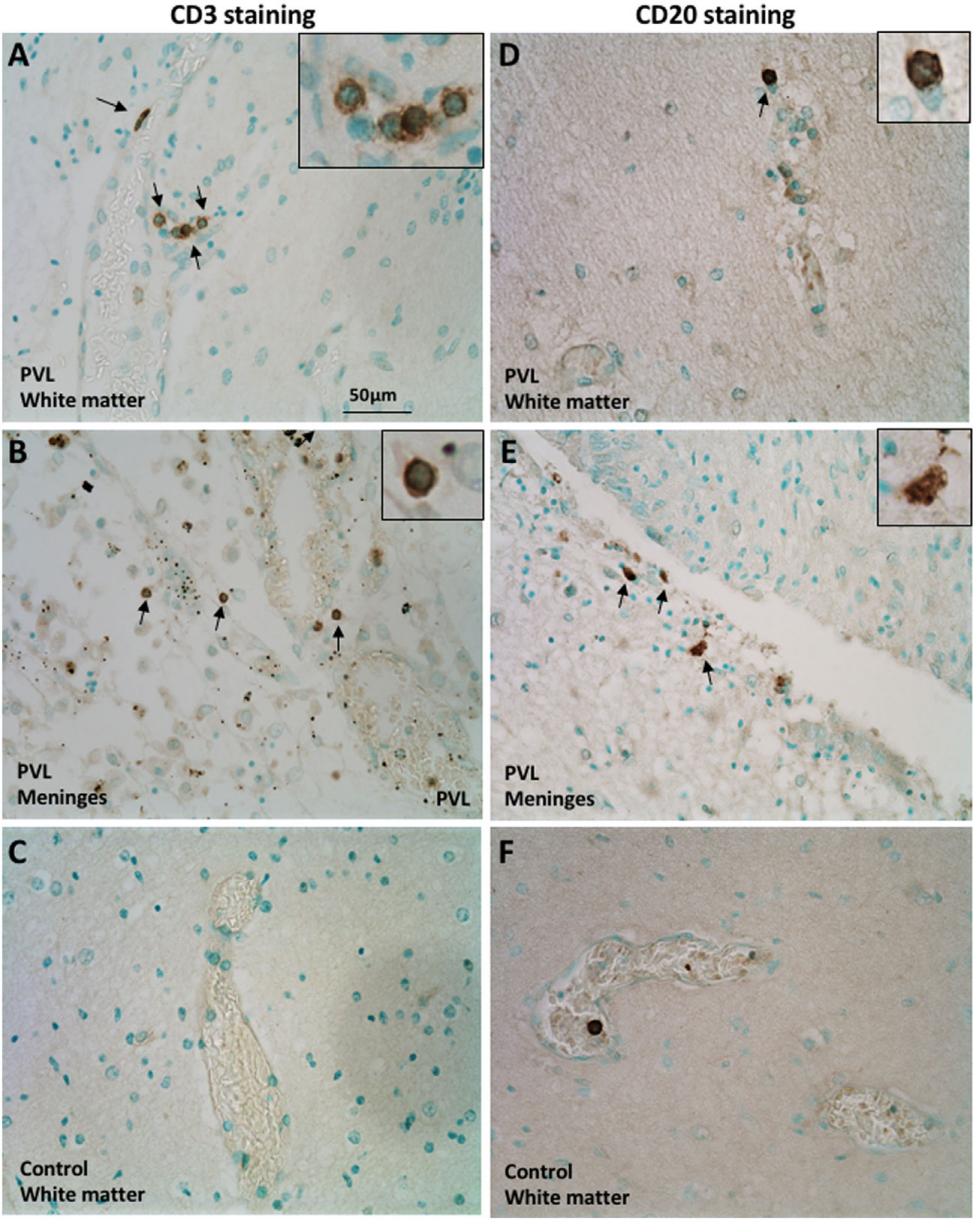
Presence of CD3^+^ T cells and CD20^+^ B cells in postmortem preterm infant brains with periventricular leukomalacia (PVL). **(A–C)** CD3^+^ T cell immunohistochemical staining in the periventricular white matter **(A,C)** and meninges **(B)** of brain sections from postmortem preterm infant brains with (case 4) and without (case 1) PVL. **(D–F)** CD20^+^ B cell immunohistochemical staining in the periventricular white matter **(D,F)** and meninges **(E)** of brain sections from postmortem preterm infant brains with (case 5) and without (case 3) PVL. Positively stained cells are indicated by arrowheads. The inserts show a higher magnification of positively stained cells.

The CD3 molecular complex is the coreceptor of the T cell receptor (TCR) and consists of five subunits—a, γ, δ, and two ε chains—and antibodies against the ε chains are often used as pan T cell markers. Immunohistochemistry on postmortem brain sections from preterm infants with PVL showed a number of CD3^+^ T cells in the periventricular white matter and meninges (Figures 1A,B). There was a higher frequency in the meninges, including cells outside the blood vessels and cells in the inner layer of the blood vessels (Figure 1B; Table 1). Such positive staining for CD3 was not observed in control cases that did not have PVL (Figure 1C).

CD20^+^ (a B cell-specific surface antigen) cells were present in the periventricular white matter in all PVL cases, albeit in small numbers (Figure 1D). B cells were also observed in the meninges of PVL cases (Figure 1E). In control cases that did not have PVL, the immunohistochemistry staining either did not reveal CD20-positive staining or the CD20^+^ cells were only observed within the blood vessels (Figure 1F).

### CD3^+^ T Cells Were Found in the Mouse Brain After HI-Induced Preterm Brain Injury

Hypoxia–ischemia triggers inflammatory processes leading to the infiltration of different immune cells at different times after injury. T cell recruitment and activation has been shown to play an important role in cerebral ischemia–reperfusion injury in adults (21, 22), but whether this is true for neonates remains unknown.

**Figure 2:**
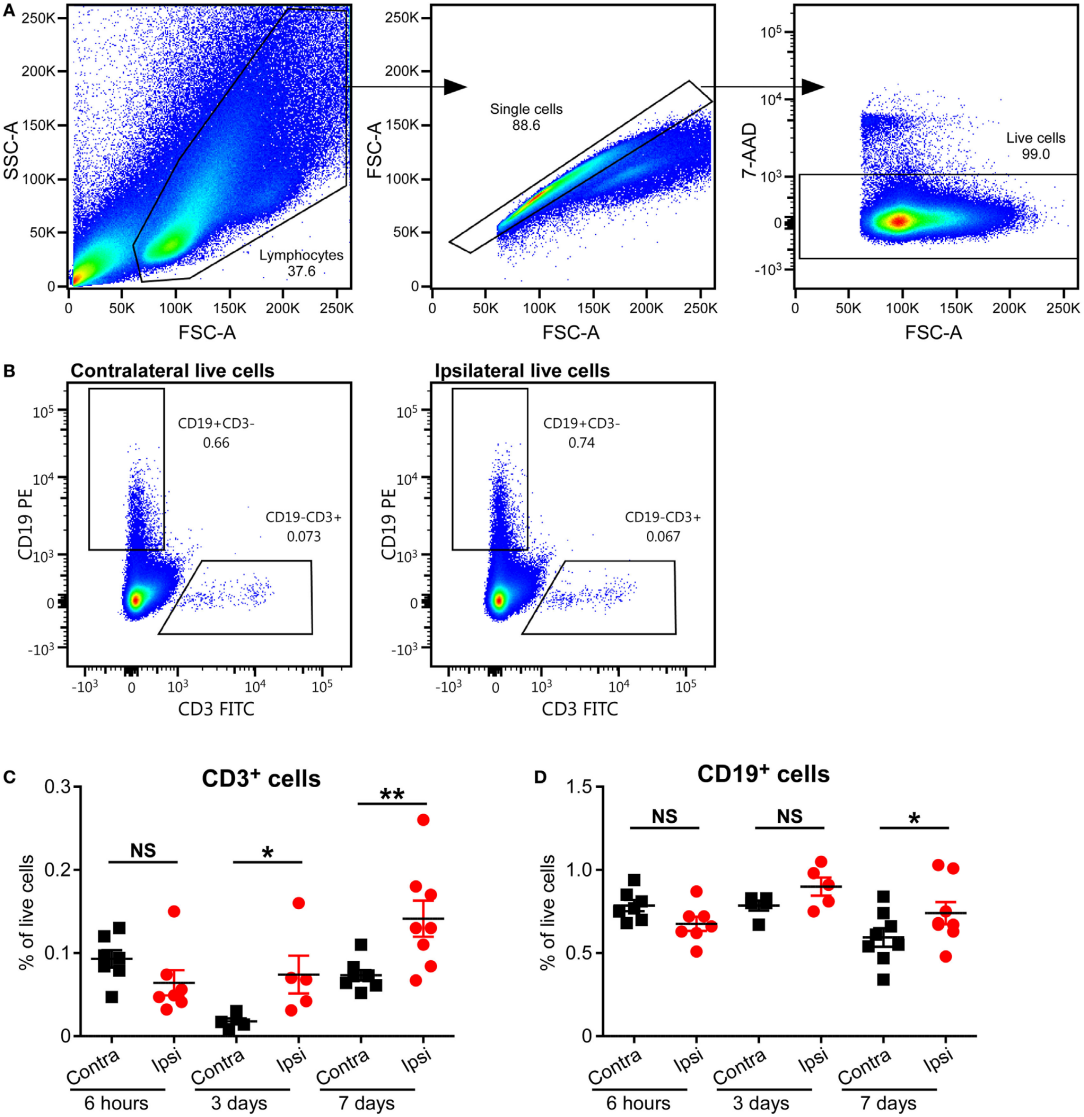
Presence of CD3^+^ T and B cells in the mouse brain after hypoxia–ischemia (HI). **(A,B)** Representative flow cytometry plots showing the gating strategy **(A)** and presence of CD3^+^ T cells and CD19^+^ B cells in the contralateral and ipsilateral hemispheres **(B)** at 7 days after HI. The percentage of CD3^+^ T **(C)** and CD19^+^ B **(D)** cells within the live cell population in the ipsilateral (Ipsi) and contralateral (Contra) hemispheres of mouse brains at 6 h (*n* = 6), 3 days (*n* = 5), and 7 days (*n* = 8) post-HI. *p < 0.05, **p < 0.01 using Student’s paired *t*-test. Data are presented as the mean ± SEM.

To study the presence of T cells in the brain, we used an established neonatal mouse model of HI-induced brain injury at a developmental age (PND5) where the brain development in the animals corresponds to that of preterm human infants (15), and we performed flow cytometry on MNCs isolated from the brains of mice subjected to HI. Significant increases in CD3^+^ T cells in the ipsilateral (injured) hemispheres compared to the contralateral (uninjured) hemispheres were observed at 3 days (*p* = 0.05) and 7 days (*p* = 0.01), but not at 6 h, after HI (Figures 2A–C).

### Increase in B Cell Frequency in the Mouse Brain Long After HI

A recent study has implicated B cells as one of the causal factors of cognitive impairments after adult stroke (23), but the role of B cells in neonatal preterm brain injury has not been studied. We used staining for CD19, the coreceptor of the B cell receptor, and flow cytometry to determine whether B cells were present in the neonatal mouse brain after HI-induced injury. The frequency of B cells was only significantly higher in the ipsilateral hemisphere compared to the contralateral hemisphere at 7 days (*p* = 0.0095), and not at 6 h or 3 days after HI (Figures 2A,B,D; Figure S1 in Supplementary Material).

### Increase in αβT Cell Frequencies in the Mouse Brain Long After HI

**Figure 3:**
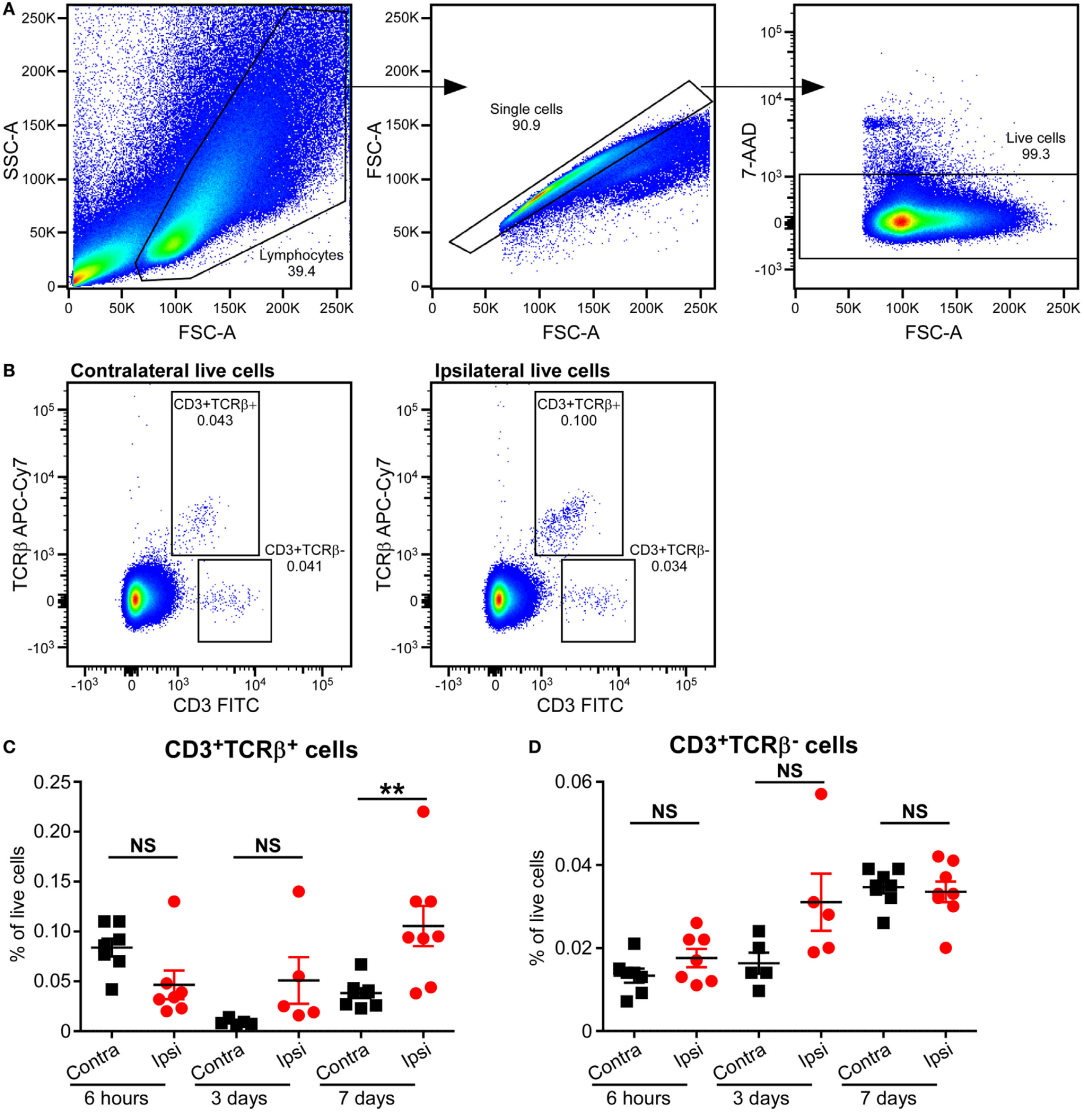
Increased CD3^+^TCRβ^+^ T cells in the mouse brain after hypoxia–ischemia (HI). **(A,B)** Representative flow cytometry plots showing the gating strategy **(A)** and presence of CD3^+^TCRβ^+^ and CD3^+^TCRβ^−^ cells in the contralateral and ipsilateral hemispheres **(B)** 7 days after HI. **(C,D)** The percentage of CD3^+^TCRβ^+^ **(C)** and CD3^+^TCRβ^−^ **(D)** cells within the live cell population in the ipsilateral (Ipsi) and contralateral (Contra) hemispheres of mouse brains at 6 h (*n* = 6), 3 days (*n* = 5), and 7 days (*n* = 8) post-HI. **p < 0.01 using Student’s paired *t*-test. Data are presented as the mean ± SEM.

Based on the type of TCR expressed on their surface, T cells are divided into αβT and γδT cells. The majority of T cells in mammals express conventional αβ TCRs on their surface, through which they recognize peptide antigens presented on MHC molecules by antigen-presenting cells. Flow cytometric analysis of mouse brains at 6 h, 3 days, and 7 days post-HI showed increased frequency of αβT (CD3^+^ TCRβ^+^) cells in the ipsilateral hemisphere compared to the contralateral hemisphere at 7 days post-HI (*p* = 0.036). No change in αβT ells was observed in the ipsilateral hemisphere at 6 h after HI (Figures 3A–C; Figure S2 in Supplementary Material).

Interestingly, A small population of CD3^+^ TCRβ^−^ cells was identified at 6 h, 3 days, and 7 days after HI. However, there was no significant difference between ipsilateral and contralateral frequency at any of the time points (Figures 3A,B,D; Figure S2 in Supplementary Material).

Neonates have reduced capacity for mounting conventional αβT cell responses due to the immature status of the αβT cells. However, γδT cells are already functionally competent during early development and are important in early life immunity. We thus evaluated the presence of γδT cells in the neonatal mouse brain at different time points after HI.

Flow cytometry analysis of MNCs isolated from brains at 6 h, 3 days, and 7 days after HI showed that there was a small population of γδT cells in the brains of naive mice, as well as in both the ipsilateral and the contralateral hemispheres of HI mice at all time points examined. However, no difference in the percentage of γδT cells was observed between the ipsilateral and contralateral hemispheres at any time points examined or between the naive animals and the animals exposed to HI (data not shown).

### Lack of Mature T and B Cells Protected Against HI-Induced White-Matter Injury

The recombination-activating gene 1 (*Rag1*) and *Rag2* genes encode the V(D)J recombinases Rag1 and Rag2 that are responsible for the rearrangement of antigen-receptor genes during T cell and B cell development. *Rag1*^−/−^ mice do not produce mature T or B cells (24), thus they are ideal for studying the effect of those immune cells in brain injury.

**Figure 4:**
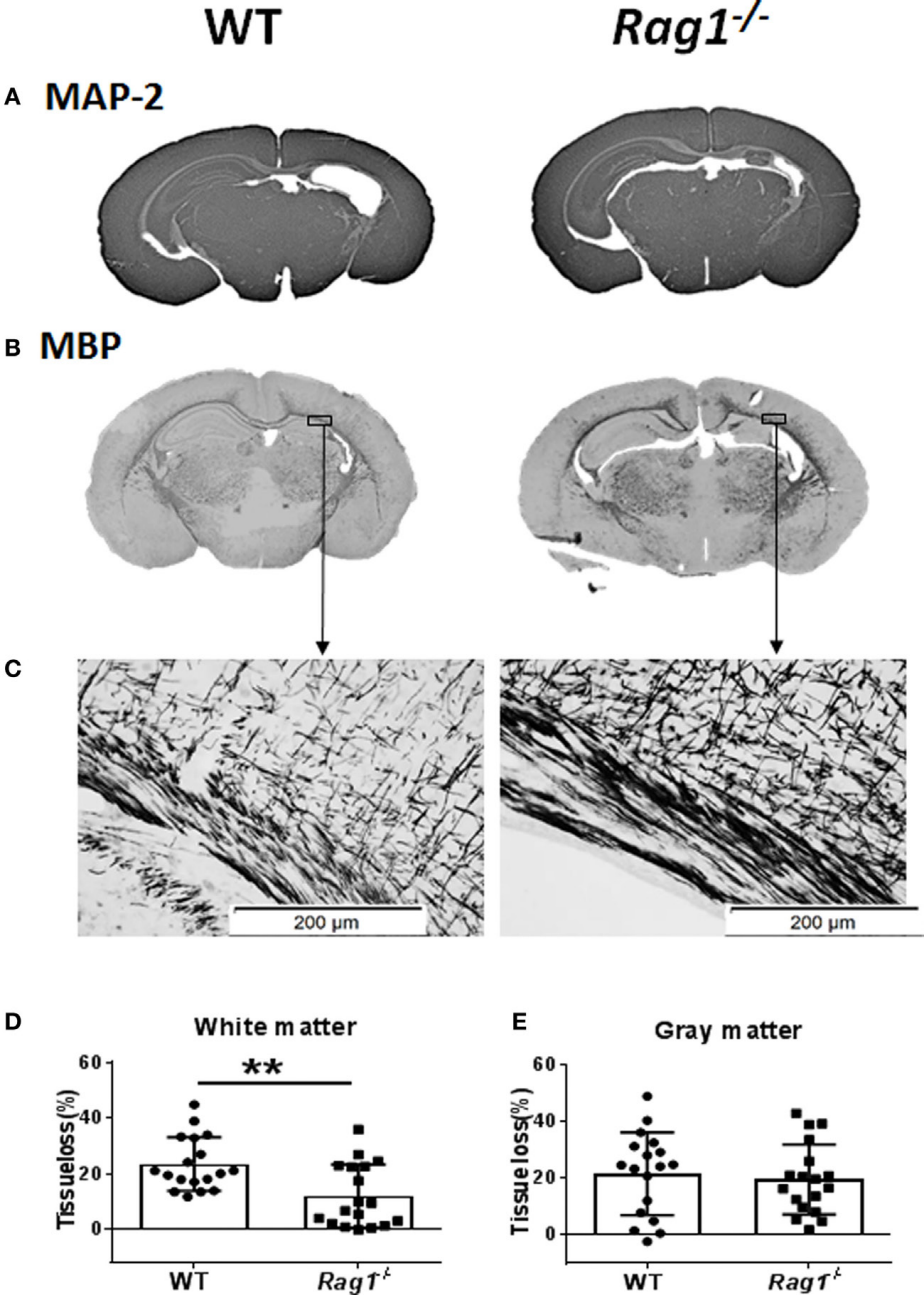
Rag1^−^**^/^**^−^ mice are protected from hypoxia–ischemia (HI)-induced white-matter brain injury. **(A,B)** Representative images of MAP2 **(A)** and myelin basic protein (MBP) **(B)** immunostainings in wild-type (WT) and *Rag1*^−^**^/^**^−^ mouse brain sections at 7 days after HI. **(C)** Higher magnification images of MBP staining in the subcortical white matter areas in the ipsilateral hemisphere. **(D,E)**, Quantification of the MBP and microtubule-associated protein 2 (MAP-2) staining shows the percentage of tissue loss in the subcortical white matter **(D)** and gray matter **(E)** in WT (*n* = 18) and *Rag1*^−^**^/^**^−^ mice (*n* = 18) at 7 days after HI insult. **p < 0.01 by Student’s unpaired *t*-test. Data are presented as the mean ± SEM.

To understand the involvement of mature T and B cells in HI-induced preterm brain injury, HI was induced in *Rag1*^−/−^ and WT mice. The extent of gray-matter injury (as indicated by MAP-2 staining, Figure 4A) and white-matter injury (as indicated by MBP staining, Figure 4B) was measured. At 7 days after HI (PND12) when myelination was formed and was visible by MBP staining, there was a significant reduction in total tissue loss in the subcortical corpus callosum white matter of *Rag1*^−/−^ mice (11.90 ± 2.67%) compared to WT mice (23.43 ± 2.27%) (*p* = 0.0023) (Figures 4C,D). Accordingly, in the subcortical corpus callosum and in the cortex of the ipsilateral hemisphere of brain sections, *Rag1*^−^*^/^*^−^ mice showed more organized and denser myelin structure compared to WT mice (Figures 4B,C) indicating reduced HI-induced white-matter injury in the Rag1^−/−^ mice compared to the WT mice.

The extent of injury in the gray-matter hemispheres of WT and *Rag1*^−^*^/^*^−^ mice was very similar (Figure 4A), and no significant difference in the total gray-matter tissue loss was observed in *Rag1*^−^*^/^*^−^ compared to WT mouse brains (Figure 4E). Further analysis of the most severely injured part of the mouse brain showed that the total tissue loss in the hippocampus area was also not significantly different between the WT and *Rag1*^−^*^/^*^−^ mice (58.1 ± 10.43% in WT mice versus 67.66 ± 6.998% in the *Rag1*^−^*^/^*^−^ mice, *p* = 0.3543).

## Discussion

The present study evaluated the potential contribution of lymphocytes to neonatal brain injury. We have provided evidence for the presence of T and B cells in the mouse brain following HI-induced neonatal brain injury. T and B cells were also present in the brains of postmortem human preterm infants with PVL; moreover, we showed that mice lacking mature T and B cells have reduced HI-induced white-matter injury.

Lymphocytes, particularly T and B lymphocytes, have been implicated in the pathogenesis of ischemic brain injury. CD4^+^ and CD8^+^ T cells have been shown to contribute to the inflammatory response, brain injury, and subsequent neurological deficits associated with adult experimental stroke (3, 21, 22). B cells have also been shown to be involved in the cognitive impairments secondary to ischemic stroke, and activated B cells infiltrate the infarct tissue adjacent to the lesion in the weeks after stroke where they undergo class switching (23). Infiltrating lymphocytes, especially T cells, can contribute to ischemic brain injury through direct damage to neurons *via* secretion of granules and cytokines, as well as through the activation of microglia, neutrophils, and brain endothelial cells (25). The circumventricular organs and perivascular spaces have been suggested as the probable route of peripheral immune cell infiltration (17, 26). Studies using both mouse and human poststroke autopsy samples have suggested the choroid plexus as the key cerebral invasion route for T cells after stroke (27).

T cells and other peripheral immune cells are found in the neonatal rodent brain following HI and persist for hours to months postinjury (12–14, 28). Consistent with these findings, our present study has shown that CD3^+^ T cells were found in the neonatal mouse brain after HI injury, and the frequency of these cells gradually increased in the days following HI. We have also shown that αβT cells and B cells were present in the injured brain at relatively late stages of the immune response.

Due to their innate-like nature and site of residence, γδT cells are involved in early immune responses against pathogenic insults in tissues (29, 30). In our recent publication on the contribution of γδT cells to neonatal brain injury, we found that γδT cells were present in the injured hemisphere as early as 6 h after injury, and mice deficient in γδT cells were protected from HI-induced (28) and sepsis-induced (11) brain injury. Different from the adult mice (31, 32), the protection we observed in the neonatal mice was IL-17 and IL-22 independent (28).

In the present study, using flow cytometry, we were able to detect a small population of γδT cells in the mouse brain, but no significant differences were observed between the naive and HI-injured brain nor between the ipsilateral and contralateral hemispheres at any of the time points examined. This might be due to the fact that γδT cells were observed in the brain meninges in both the neonatal and adult mice (28, 33); therefore, using whole brain homogenate to run flow cytometry is not a sensitive method for detecting the differences in this relatively small γδT cell population.

In contrast to the γδT cells, we found that the frequency of αβT cells increased in the injured neonatal brain at late time points (7 days) after HI. This is in line with previous findings in rodent models of neonatal HI brain injury and in adult experimental stroke (12–14). Conventional αβT cells start to infiltrate the injured brain as early as 3 days after stroke in mice (3). A similar study showed CD4^+^ T cell infiltration in the infarct region starting 7 days after ischemia, and the number of T cells peaked 14 days postinjury (34). Taken together, our findings and those of others suggest that conventional αβT cells are involved in immune responses at late time points after brain injury in both neonatal and adult mice.

We observed the presence of CD3^+^ TCRβ^−^ cells in the neonatal HI brain at all assessed time points. These cells could be natural killer T (NKT) cells or CD4 and CD8 double-negative T cells. NKT cells have been shown to play a role in ischemic reperfusion brain injury, and especially in the associated systemic bacterial infection (35). However, the role of CD4 and CD8 double-negative T cells in adult and neonatal ischemia injury has not been studied until now. Despite non-significant changes in this population in the current study, future studies investigating the role of these CD3^+^ TCRβ^−^ cells in neonatal HI brain injury could provide further understanding of the role of lymphocytes in this injury model.

Using flow cytometry, we observed the presence of a small but clear B cell population in the mouse brain after HI. The frequency of these cells was significantly increased in the ipsilateral hemisphere 7 days after injury when compared to the contralateral hemisphere. In adults, activated B cells infiltrate the infarct tissue in the weeks following stroke, and lack of B cells prevents the appearance of delayed cognitive deficits caused by ischemia (23). In contrast, a recent study has shown that B cells do not have a major pathophysiological role in acute ischemic stroke in mice (36). To better understand the role of B cells in neonatal brain injury, further studies are needed.

Using immunohistochemistry, we have identified the presence of CD3^+^ T cells, and to a smaller extent B cells, in the postmortem brains of preterm infants. Despite the limitation of the total number of clinical cases used in this study, the findings were consistent and correlated with the mouse data. Therefore, the human postmortem preterm samples served as a valuable complimentary addition and strengthened the conclusions of the current study. Together with our other recent publication (28), we provide evidence for the presence of lymphocytes in the human postmortem brain of preterm infants with PVL. These results demonstrate a clear requirement for further studies using clinical cases to better characterize the role of these immune cells in neonatal brain injury.

Our *Rag1*^−^*^/^*^−^ data demonstrated that the lack of T and B cells protects against HI-induced preterm brain injury. *Rag1*^−^*^/^*^−^ mice showed reduced white matter brain tissue loss compared to WT mice at 7 days after HI. This finding is consistent with previous adult stroke studies, where mice lacking both T and B cells have significant reductions in brain injury (21, 22). Despite the potential contribution of innate immune cells in *Rag1*^−/−^ mice to the protection against brain injury (37–39), our studies and others support the critical role of T and B cells in ischemic brain injury in both immature and mature developmental stages.

The protective effect of T and B cells deficiency is only observed in the white matter and not in the gray matter of HI mice. This is an interesting finding and warrants further investigations in the future.

The lack of molecular mechanisms behind the functional regulation of T and B lymphocytes, as well as long-term anatomical and functional outcomes, are some of the limitations of the current study. Furthermore, brain injury could have been more thoroughly evaluated by a full stereological assessment.

In summary, our study provides a temporal analysis of the presence of different populations of lymphocytes in a neonatal mouse model of preterm brain injury. Together with our other most recent publications, we have provided a description of the immune response (15) and the presence of lymphocytes (28), especially γδT cells (11, 28), in the mouse and human brain after preterm brain injury. Our current observation that mature lymphocyte deficiency protects neonatal mice from HI-induced brain white matter injury provides new evidence for the mechanism of neonatal brain injury that will help in the development of therapeutic strategies for such conditions.

## Ethics Statement

National Research Ethics Service, Hammersmith and Queen Charlotte’s and Chelsea Research Ethics Services, London, UK (ethics number 07/H0707/139). Animal Ethical Committee of the University of Gothenburg, Sweden (ethical number 5/2013 and 58-2016).

## Author Contributions

XW, JWL, AN, and A-MA conceived and designed the study; AN, A-MA, XZ, ER-F, JL, AZ, and RV performed the experiments and analyzed the data; AN drafted the manuscript. XW, JL, ER-F, JWL, and CM revised the manuscript. MR, CZ, CM, HH and GN revising it critically for important intellectual content, and provide approval for publication of the content. All authors contributed to data interpretation and approved the submitted version.

## Conflict of Interest Statement

The authors declare that the research was conducted in the absence of any commercial or financial relationships that could be construed as a potential conflict of interest.

## Supplementary Material

Figure S1 Gating strategy of CD19 B cells in the mouse brain after hypoxia–ischemia (HI). **(A)** Representative flow cytometry plots showing the gating strategy of CD3^−^ CD19^+^ events in the brain at 6 h after HI. **(B)** The CD3^−^ CD19^+^ population backgated on CD11b versus CD45 plot to ensure a true population was detected and not myeloid cells with insufficient blocking of Fc receptors. **(C)** Fluorescent minus one (FMO) controls for CD19 staining.

Figure S2 Gating strategy of T cells in the mouse brain after hypoxia–ischemia (HI). **(A)** Representative flow cytometry plots showing the gating strategy of CD3^+^ TCRβ^+^ events in the brain at 6 h after HI. **(B)** The CD3^+^ TCRβ^+^ population backgated on CD11b versus CD45 plot to ensure a true population was detected and not myeloid cells with insufficient blocking of Fc receptors. **(C)** Fluorescent minus one (FMO) controls for CD3 and TCRβ staining.
