# Supplementary figures and images for: Lymphocytes Contribute to the Pathophysiology of Neonatal Brain Injury
Source: Front Neurol. 2018 Mar 19;9:159. doi: 10.3389/fneur.2018.00159 (PMC5868390; doi:10.3389/fneur.2018.00159)

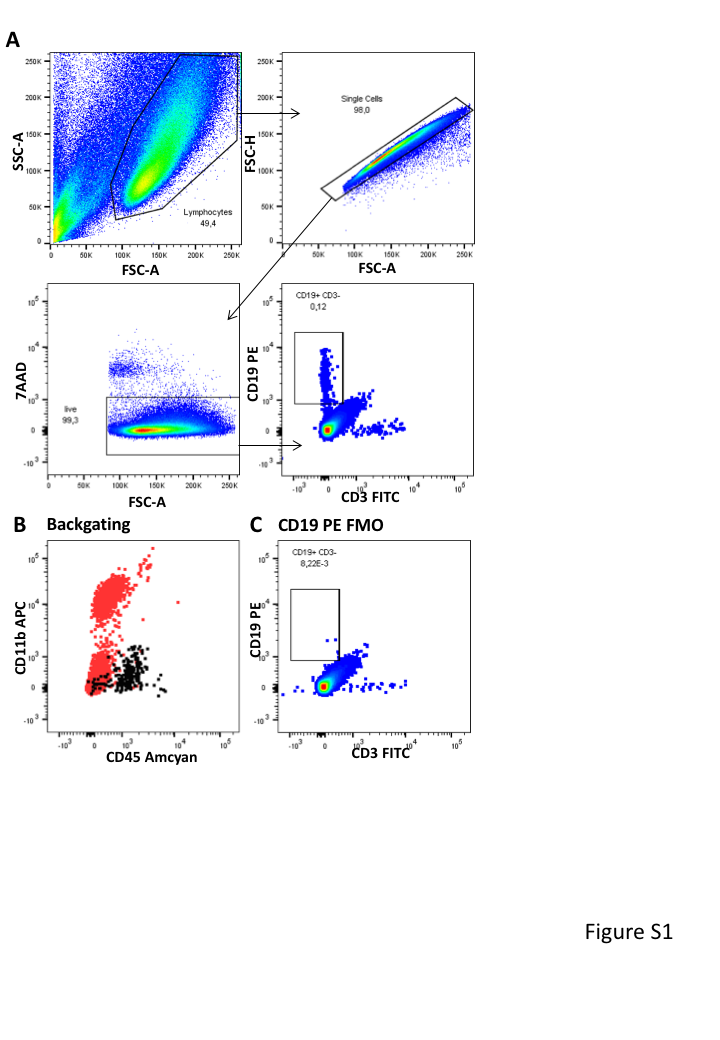

Supplement: Figure S1 — Gating strategy of CD19 B cells in the mouse brain after hypoxia–ischemia (HI). (A) Representative flow cytometry plots showing the gating strategy of CD3− CD19+ events in the brain at 6 h after HI. (B) The CD3− CD19+ population backgated on CD11b versus CD45 plot to ensure a true population was detected and not myeloid cells with insufficient blocking of Fc receptors. (C) Fluorescent minus one (FMO) controls for CD19 staining. [file Image_1.TIFF]

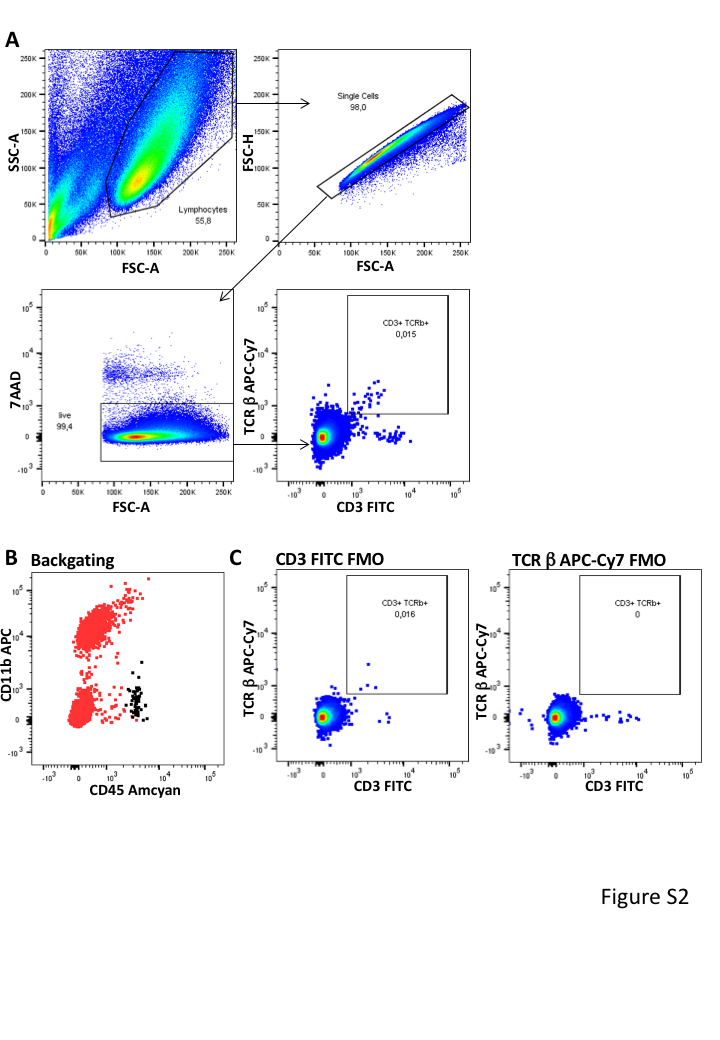

Supplement: Figure S2 — Gating strategy of T cells in the mouse brain after hypoxia–ischemia (HI). (A) Representative flow cytometry plots showing the gating strategy of CD3+ TCRβ+ events in the brain at 6 h after HI. (B) The CD3+ TCRβ+ population backgated on CD11b versus CD45 plot to ensure a true population was detected and not myeloid cells with insufficient blocking of Fc receptors. (C) Fluorescent minus one (FMO) controls for CD3 and TCRβ staining. [file Image_2.TIFF]
